# Risk Assessments of Patients Admitted to Mixed Inpatient Psychiatric Wards: A Clinical Audit

**DOI:** 10.1192/bjo.2022.429

**Published:** 2022-06-20

**Authors:** Sharna Bennett, Vijay Delaffon

**Affiliations:** Kent and Medway NHS and Social Care Partnership NHS Trust, Kent, United Kingdom

## Abstract

**Aims:**

Following a Serious Incident (SI) on a mixed sex ward; it was important to investigate whether this is a widespread problem in Psychiatry. The acute care group standard is that patients with known risk to the opposite sex should not be admitted to mixed sex wards. A comprehensive risk assessment should take place when a patient is admitted to a mixed sex ward. Furthermore, if any risks are identified, these should be escalated to the multidisciplinary team (MDT), including the nurse-in-charge and on-call Consultant Psychiatrist.

**Methods:**

We conducted a literature search to establish how different Trusts consider risk when arranging for admission, as well as to identify whether single-sex wards have helped to reduce the incidence of serious incidents. We then retrospectively collected data from 10 inpatients present on mixed sex wards throughout Kent and Medway in May 2021. This involved searching electronic notes at the point of admission, including progress notes and risk assessments to identify whether information is present to suggest that an admission to a mixed sex ward is unsuitable, and if so, whether this has been appropriately escalated.

**Results:**

When patient notes were surveyed, only 50% of patients had a full risk assessment documented. Historical risks were documented in 40% of patients notes at admission. Junior doctors are required to complete an admission clerking for new patients, which should include a risk assessment; 70% of these contained a risk assessment, and 60% discussed risks towards others. 30% of patients had identifiable risks to the opposite sex but were admitted to a mixed sex ward. However, none of these cases were escalated to the MDT for discussion regarding the most suitable ward for the patient.

**Conclusion:**

When patients are admitted to any inpatient psychiatric ward it is important to document a full risk assessment including historical risks. Unfortunately, full risk assessments were not always carried out at the point of admission, meaning that patients who had been admitted to mixed sex wards remained there despite previously documented risks. In general, junior doctors included risk assessments in their admission clerkings, but there is evidently room for improvement from all healthcare professionals. Recommendations for improvement are to generate specific guidance for documenting risk assessments and to offer teaching to healthcare professionals on ensuring they have completed a comprehensive risk assessment and when it is appropriate to escalate this to ensure further serious incidents do not occur. Re-audit is scheduled for March 2022.

